# Translation inhibitors cause abnormalities in ribosome profiling experiments

**DOI:** 10.1093/nar/gku671

**Published:** 2014-07-23

**Authors:** Maxim V. Gerashchenko, Vadim N. Gladyshev

**Affiliations:** Division of Genetics, Department of Medicine, Brigham & Women's Hospital and Harvard Medical School, Boston, MA 02115, USA

## Abstract

Ribosome profiling and high-throughput sequencing provide unprecedented opportunities for the analysis of mRNA translation. Using this novel method, several studies have demonstrated the widespread role of short upstream reading frames in translational control as well as slower elongation at the beginning of open reading frames in response to stress. Based on the initial studies, the importance of adding or omitting translation inhibitors, such as cycloheximide, was noted as it markedly affected ribosome coverage profiles. For that reason, many recent studies omitted translation inhibitors in the culture medium. Here, we investigate the influence of ranging cycloheximide concentrations on ribosome profiles in *Saccharomyces cerevisiae* and demonstrate that increasing the drug concentration can overcome some of the artifacts. We subjected cells to various manipulations and show that neither oxidative stress nor heat shock nor amino acid starvation affect translation elongation. Instead, the observations in the initial studies are the result of cycloheximide-inflicted artifacts. Likewise, we find little support for short upstream reading frames to be involved in widespread protein synthesis regulation under stress conditions. Our study highlights the need for better standardization of ribosome profiling methods.

## INTRODUCTION

**Figure 1.**
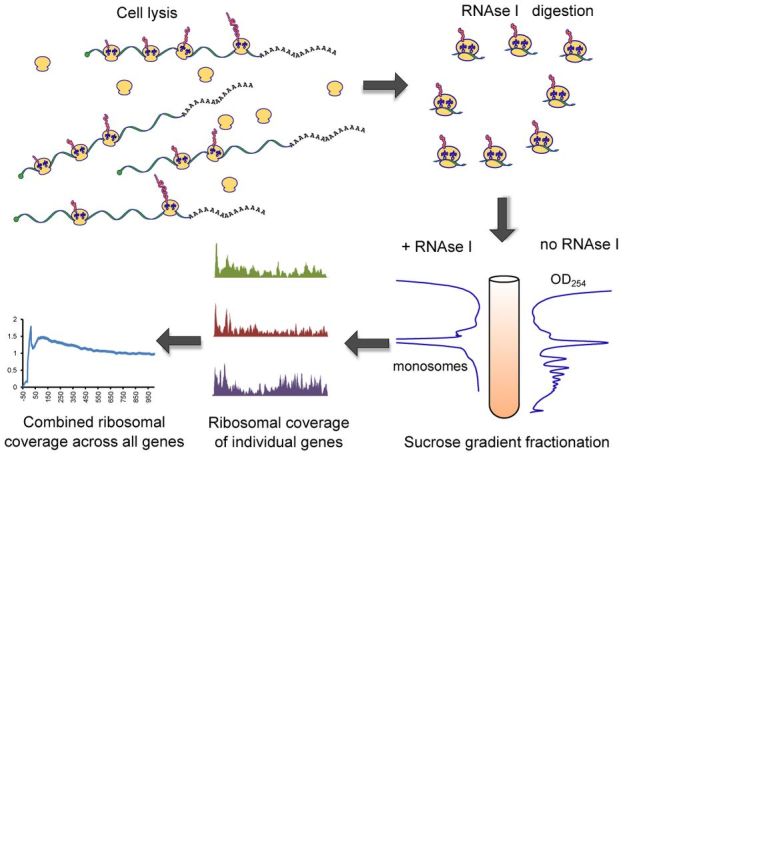
Ribosome profiling. Cell lysis releases a mixture of individual ribosomal subunits, assembled ribosomes in complex with mRNA and blank ribosomes with no RNA attached. Sucrose gradient fractionation allows separation and isolation of these components. Captured mRNA fragments are then sequenced on an Illumina platform.

Ribosomal profiling is a common designation for several methods that examine translation *in vivo* by characterizing mRNA transcripts engaged in interaction with active ribosomes. A key advance in this approach has recently been made by isolating mRNA fragments (‘footprints’) from actively translating ribosomes and subjecting them to high-throughput sequencing (Ribo-seq) (1). The footprints reveal the positions within mRNA occupied by translating ribosomes, allowing genome-wide quantification and analysis of translation at the level of genes and codons. In most cases, the exact codon in either A or P site of the ribosome can be determined because footprints have uniform length distribution (Figure 1).

Recent explosion of interest in the use of Ribo-seq to address numerous questions related to translation proved a remarkable potential of this method. Several Ribo-seq studies reported novel and unexpected features of protein synthesis in yeast and mammals. For example, the ribosome distribution along mRNA was not uniform: there was a larger fraction of ribosomes residing at the beginning of transcripts, 100–200 nucleotides downstream of the start codon in yeast, pointing to slower elongation in this region. Another novel feature attributed to translational control was the widespread use and highly increased ribosomal occupancy at short upstream open reading frames (uORF) in response to amino acid starvation (1). A study from our group showed a similar outcome under conditions of oxidative stress (2). It was also reported that ribosomal occupancy increases immediately downstream of the start codon as a function of heat shock stress in mammalian cell cultures (3). However, shortly after introducing Ribo-seq, some concerns have been raised regarding ribosome distribution on mRNA. It was suggested, that peaks of footprint densities is a result of cycloheximide-inflicted accumulation of ribosomes, when the drug is added to growing cell culture (4). In yeast, when the drug is not supplemented until cell lysis, the peaks were significantly lower, and there was not much difference in mammalian cells (5). In these studies, a side-by-side comparison of cycloheximide effects were done on unstressed cells so it leaves a question open as to how persistent these artifacts when the stress is taken under consideration.

In this study, we investigated how translation inhibition distorts footprint coverage across mRNA transcripts and demonstrated that the intensity of ribosome accumulation strongly depends both on the intensity of stress and the concentration of cycloheximide. We found no evidence of translation elongation being affected by various stress types in Ribo-seq studies.

## MATERIALS AND METHODS

Extended material and methods can be found in Supplementary Information.

### Yeast strains and growth conditions

*Saccharomyces cerevisiae* strain BY4741 was grown on YPD (Yeast extract, peptone, dextrose) agar plates for several days prior to experiments. Unless otherwise stated, the day before the experiment cells were transferred to a 50 ml flask of YPD medium and grown overnight at 30°C with shaking. A part of that culture was inoculated into 500 ml of fresh YPD at the initial OD_600_ = 0.025 and incubated at 30°C with shaking until the OD_600_ reached 0.5–0.6. If cultures were designated for cyloheximide treatment, the drug was added at the end of any additional stress-inducing incubation. Immediately after drug addition, cells were harvested by vacuum filtration on 65 um polyvinylidene difluoride (PVDF) filters (Millipore). It took exactly 5 min to collect the cells, which then were snap frozen in liquid nitrogen. If no drug treatment was needed, yeast cells were collected in the same manner, but filtration was initiated 5 min before the stress had to finish.

### Cycloheximide treatment

Concentrations of cycloheximide ranging from 1.56 to 10,000 μg/ml were used. We refer to 100 μg/ml as ‘x1’, because it was used to inhibit translation in all other studies cited in this report. Therefore, other concentrations were marked as x1/64, x1/16, x1/4, x8, x100. To achieve x8 concentration, we prepared the stock solution in dimethyl sulfoxide (DMSO). The highest possible concentration x100 was the most challenging. We first collected yeast cells by filtration, rapidly resuspended them in 5 ml of filtered YPD medium and added 5 ml of YPD with 20 mg/ml cycloheximide. This is the highest concentration possible considering drug solubility in water-based solvents. Stress conditions are listed in the Supplementary Information.

### Cell lysis and ribosome isolation

Frozen cell paste pellets were pulverized in a Mini Bead Beater (BioSpec) using stainless steel vials and chromium beads. To prevent yeast thawing, pulverization was done in multiple 10 s cycles where vials were repeatedly submerged to liquid nitrogen after each cycle. Therefore, the content of vials was kept frozen during pulverization. Note that 1 ml of ice cold lysis buffer (20 mM Tris-HCl pH 8.0, 140 mM KCl, 5 mM MgCl_2_, 1% Triton-X100, 100 μg/ml cycloheximide) was used to resuspend the pulverized cell powder. The lysates were clarified by centrifugation for 5 min. Note that 30 OD_260_ units of cell extract were treated with 600 units of RNAse I (Life Tech, Ambion) for 1 h at room temperature. The lysates were loaded on top of 10–50% sucrose gradient, prepared in the lysis buffer with no Triton. Ultracentrifugation in SW-41 Ti rotor for 3 h at 35 000 revolutions per minute and 4°C separated large ribosomal complexes from other cellular components. After fractionation in a sucrose gradient, the monosomal fraction was collected and footprints were isolated.

### Sequencing library preparation

An oligonucleotide adapter was ligated to the 3′ end of footprints. It served as an anchor to a reverse transcription primer. Reverse transcription was followed by circular ligation and polymerase chain reaction amplification of libraries prior to sequencing on the Illumina HiSeq 2000 platform.

### Footprint alignment

Bowtie software v. 0.12.7 (6) was used to align footprints to yeast genome (*S. cerevisiae* S288C genome, downloaded from SGD database with annotations). Custom Perl scripts were implemented to pre-process alignment files and plot ribosomal occupancy.

### Ribosomal occupancy distribution plot

We selected all single exon genes longer than 1000 nucleotides expressed at reads per kilobase per million (rpkm) > 30. They were aligned by start codon and coverage at every nucleotide position of every gene was averaged. The plot covers 1000 nucleotides within reading frame plus 50 nucleotides upstream of the start codon. The average coverage density of the last 300 nucleotides was used to normalize ribosome occupancy so that each profile approached the value of 1.0. We used the entire footprint sequence to calculate coverage, therefore, the profile line appears smooth. Alternatively, only 5′ or 3′ ends of footprints could be used, then the profile would be more irregular.

## RESULTS AND DISCUSSION

**Figure 2.**
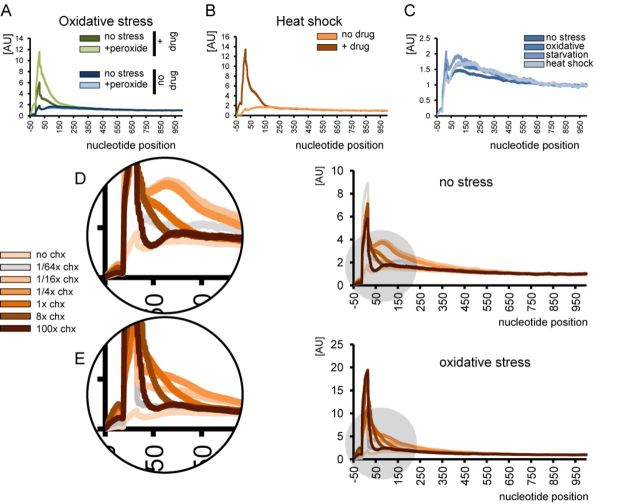
Ribosomal occupancy profiles and the effect of stress and drug treatment. (A) Control yeast cells and cells treated with hydrogen peroxide (0.2 mM) in the presence or absence of 100 μg/ml cycloheximide in the media. Nucleotide position count is relative to start codon. (B) Ribosome occupancy profiles of yeast cells undergoing heat shock (42°C, 20 min). The peak appears only when cycloheximide is added to the medium. (C) None of the three tested stress types lead to a significant increase of ribosomes at the 5′ proximities of reading frames in the absence of cycloheximide. Refer to Figure 3A and B for additional details on amino acid starvation. (D and E) Concentration of cycloheximide in the medium affects the shape of the profile, pointing to a passive diffusion model of cycloheximide entering live cells. Cells were grown in YPD medium in the absence of stress (D) or subjected to oxidative stress (0.2 mM hydrogen peroxide, 30 min) (E). Cycloheximide concentration does not immediately reach the threshold, under which all ribosomes are inhibited with 100% efficiency, instead increasing gradually. Therefore, following the treatment some ribosomes initiating translation continue protein synthesis until they encounter the drug, leading to a broad cumulative peak in the ribosomal occupancy profile.

**Figure 3.**
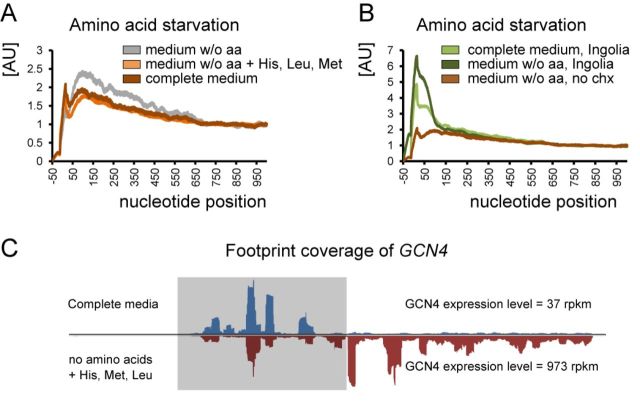
In-depth investigation of amino acid starvation and changes in the ribosomal profile. (A) Repeating the experiment as was done in (1) but without cycloheximide pre-treatment still leads to a slightly different ribosomal occupancy profiles (gray and dark brown lines on the graph). However, the yeast strain BY4741, used in that study is auxotroph in histidine, leucine and methionine, which are used as selective markers. Depletion of culture medium of all amino acids cannot be considered as starvation, because the lack of three essential amino acids will lead to cell death rather than to metabolism switching toward synthesis of its own amino acids. Therefore, we supplemented the medium without amino acids with normal levels of His, Met and Leu. As a result, the difference in ribosomal profiles between starved and non-starved conditions disappeared. Thus, amino acid starvation does not cause the accumulation of ribosomes at the beginning of ORFs or uORFs. The only scenario when this accumulation was observed is the absolute lack of the essential amino acids, leading to ribosome stalling at corresponding codons. This is a very extreme case, which has little in common with regulation *per se*. (B) Ribo-seq data published in (1) were processed with our analytical approaches (cycloheximide was present in the culture medium). Brown line is based on our experiment (same as in Figure 3A). (C) Footprint coverage of *GCN4* in response to amino acid stress derived from our experiments.

Published studies on ribosomal profiling of both yeast and mammalian cells have used somewhat different sample preparation methods, which complicate direct comparison. Therefore, we reproduced some treatments and stresses using budding yeast as a model organism. *S. cerevisiae* cells were tested for amino acid starvation (as in (1)), oxidative stress (as in (2)) and heat shock (as in (3)), with and without drug treatment in the culture medium. As expected, we observed a different distribution of ribosomal occupancy, wherein the broad peak downstream of the start codon decreased in the absence of the drug. Unexpectedly, there was no increase in response to stress (Figures 2A–C and 3). Apparently, none of the stress conditions tested targeted the translation elongation step.

To examine in more detail how the drug influences the ribosomal distribution, we performed a series of experiments with the cycloheximide concentrations ranging from 1.56 to 10 000 μg/ml in the medium. Normal and oxidative stress conditions were chosen for a side-by-side comparison. The shape of the occupancy peak was not constant (Figure 2D and E). The most important observation was the disproportionate increase in ribosomal occupancy under stress that was completely reversed by elevated concentrations of translation inhibitor. In other words, the artifactual input resulting from 100 μg/ml cycloheximide treatment was not constant and was highly dependent on stress intensity.

These observations point to a passive diffusion model of cycloheximide entering live cells. The drug diffuses in a concentration-dependent manner, e.g. it takes up to 2 min to reach the equilibrium between the ‘in’ and ‘out’ cycloheximide concentrations (7,8). The data further suggest that the cycloheximide concentration does not immediately reach the threshold, under which all ribosomes are inhibited with 100% efficiency, instead increasing gradually. Therefore, following the treatment some ribosomes initiating translation continue protein synthesis until they encounter the drug, leading to a broad cumulative peak in the ribosomal occupancy profile. The area under the peak is increased under conditions of acute stress, which leads to a steep decrease in the translation initiation rate, supposedly increasing the ratio of initiating/elongating ribosomes. Thus, the effect of drug treatment becomes more pronounced and reflects stress intensity rather than being a feature of translation (Figures 2 and 3).

Based on Ribo-seq data, several studies proposed that an enrichment of ‘slow’ codons right after the start codon is responsible for the general decrease in elongation rate (9,10). A recent *in silico* study questioned this hypothesis, but still viewed the peak of ribosomal occupancy as a feature of translation in eukaryotes (11). Our data, however, suggests complete departure from the slow translation model, as the increase in ribosomal occupancy at the 5′ proximities of genes is dramatically less prominent than previously thought. Consistent with other observations (12,13), there was only a residual slope, descending more than 300 nucleotides downstream of the start codon, implying a limited influence of a ramp of rare codons or other factors. In addition, stress does not change the ribosomal distribution (Figure 2). Therefore, we think that the degree of ribosome accumulation is not sufficient to regulate translation and does not play a notable role in stress response.

Mammalian cells typically show no broad peak in the absence of stress regardless of drug treatment (5). However, heat shock and proteotoxic stress increase ribosomal occupancy in the presence of the drug (3,14). By analogy with yeast, this could be an artifact, and additional profiling experiments should bring clarity on this issue. The reason the effect is more prominent in yeast is the presence of thick cell wall serving as an additional barrier for passive diffusion. There are certain obstacles with omitting drug treatment in mammalian cell cultures, for they typically grow attached to flask surface. Unlike rigid yeast cells, mammalian cells cannot be subjected to harsh mechanical interventions without losing cell integrity. Therefore, we recommend using a higher concentration of cycloheximide or any other translation inhibitor while performing ribosome profiling experiments in cell culture. However, since our study was conducted solely using a yeast model, we cannot be certain whether the observations made in (3,14) are artifactual. Considering similarity and conservation of translation machinery between yeast and mammals it seems reasonable to utilize Ribo-seq experiments with ranging cycloheximide concentrations to determine if the translation elongation slowdown persists or not.

**Figure 4.**
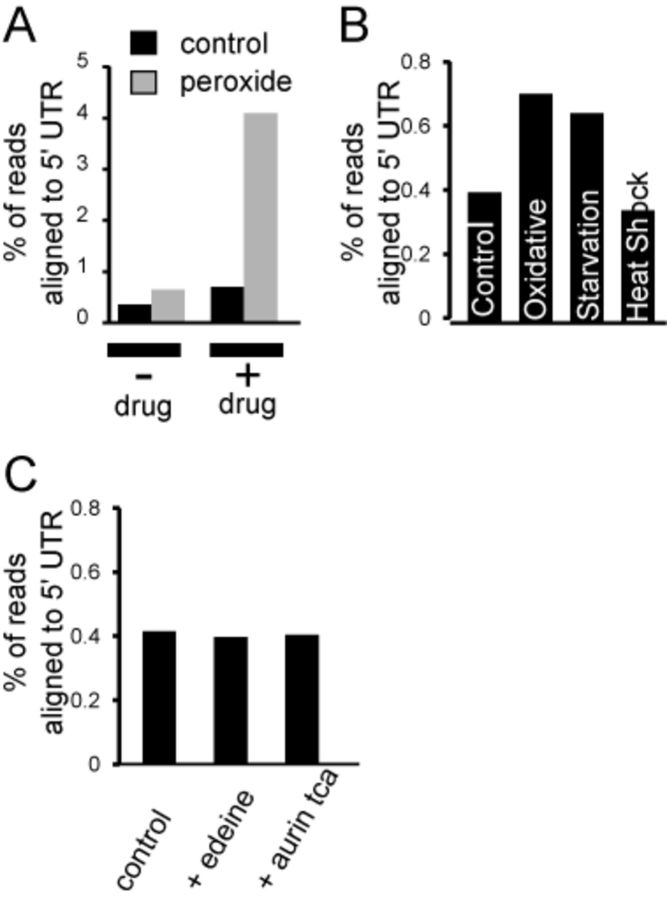
uORF and 5′ UTR coverage in ribosome profiling experiments. (A) There is a dramatic difference in cumulative 5′ UTR occupancy if cycloheximide treatment is omitted. (B) None of the three examined stresses significantly increase 5′ UTR ribosomal occupancy. Although oxidative stress and amino acid starvation do slightly increase 5′ UTR occupancy, the effect is minimal compared to what was previously found (1,2). (C) Addition of translation initiation inhibitors does not affect cumulative ribosomal occupancy at the 5′ UTR.

Another finding in our study relates to the abundance and characteristics of uORFs. It has been shown that amino acid starvation leads to a significant increase in ribosome occupancy at uORFs (1). A similar behavior was reported for meiosis and oxidative stress (2,15). However, Ribo-seq carried out without cycloheximide treatment revealed no evidence for a general increase in uORFs regardless of stress type (Figure 4A and B). A typical cycloheximide treatment provides plenty of time for free ribosomes to initiate translation and get stalled at the start codon by cycloheximide. In other words, an increase in uORF occupancy during stress co-occurs with the accumulation of footprints at the beginning of regular ORFs, it is likely artifactual and depends on stress intensity. In our opinion, there is no general trend supporting the use of uORFs in hundreds of genes in response to stress, although certain genes do depend on this type of regulation, such as *GCN4* transcription factor (Figure 3C). It should be noted, however, that the methodology of sample preparation in published studies, as well as in our current study, could also be criticized. For instance, there might be a run-off of ribosomes from uORFs the moment the lysis buffer is added to frozen pulverized cells. The rate of translation in eukaryotic cells is more than six codons per second, therefore, even a split second before the cycloheximide hits the ribosome could be sufficient to deplete footprints from uORFs. As true as it is in case of uORFs, it has to be true with regular protein-coding ORFs. On the other hand, we always observed a peak right at the start AUG codon, no matter how the sample is treated and processed. We would like to emphasize that our study does not deny the existence of multiple uORF in a yeast genome. The extent of their participation in stress response is what we think has been overestimated. We suggest stricter experimental criteria to be applied in order to consider the footprints coming from uORFs genuine. Although we favor the hypothesis that uORFs are not stress-inducible in general, the experiments presented in this study do not inevitably exclude previously published observations. Our study rather illustrates the uncertainty of Ribo-seq generated data when it comes to addressing 5′ terminal translation events.

The lysis buffer composition should not allow elongation due to a higher than optimal concentration of magnesium and presence of cycloheximide. However, it leaves the possibility that small and large ribosomal subunits spontaneously assemble at upstream AUG codons, contributing to the pool of uORF footprints. We excluded this scenario by preventing ribosomal assembly in cell lysates. Neither the presence of edeine which blocks joining of 60S and 40S subunits (16), nor aurintricarboxylic acid, a general inhibitor of protein-RNA interactions (17), in the lysis buffer affected the share of footprints from 5′ untranslated regions (UTRs) (Figure 4C). This information should be useful for those who use Ribo-seq *in vitro* for studying translation mechanics in cell-free lysates.

**Figure 5.**
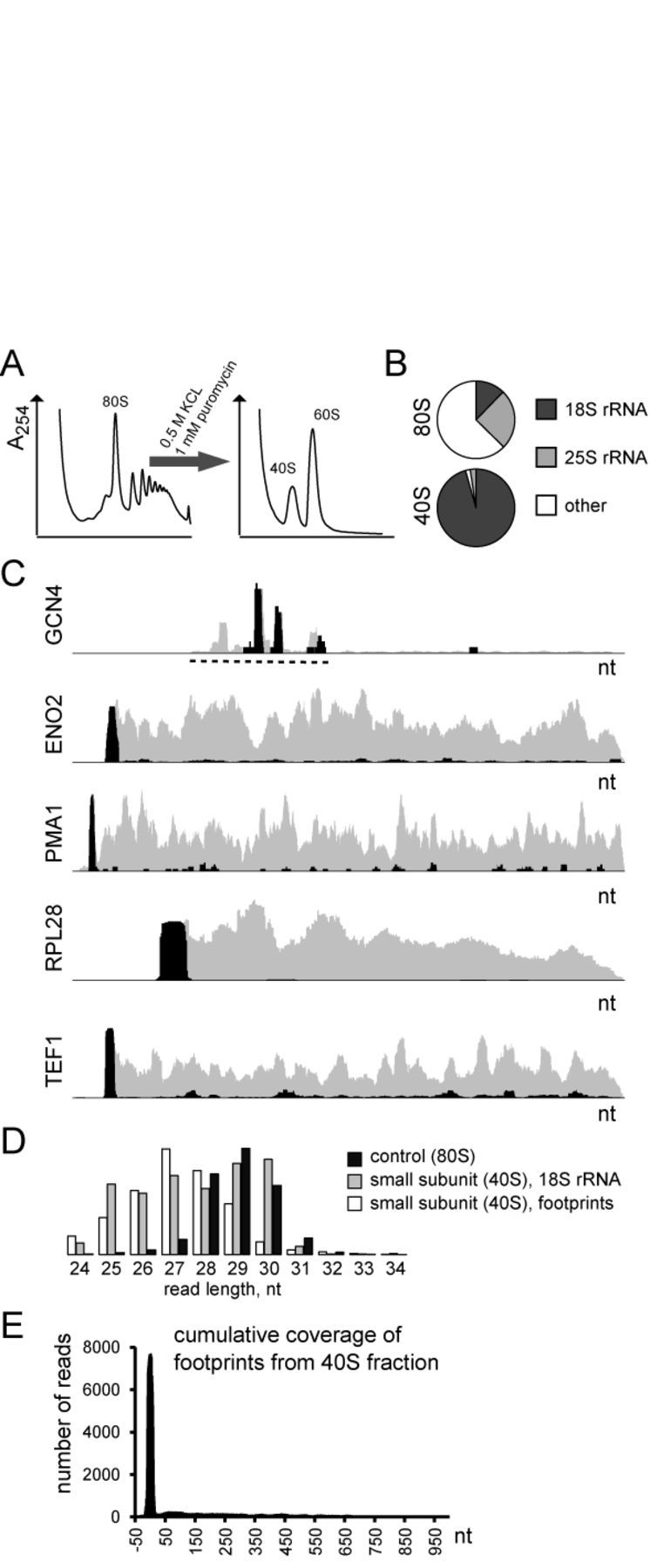
Ribo-seq of the small ribosomal subunit. (A) Dissociation of monosomes and polysomes into subunits. Fractions corresponding to the 40S small ribosomal subunit were collected for sequencing. (B) Shares of reads aligned to 18S and 25S rRNAs in Ribo-seq samples. The upper chart shows typical shares of reads in 80S fraction in control cells (YPD media, log growth phase, no stress, no cycloheximide pretreatment), and the lower shares of reads in the 40S fraction. Although not precisely quantitative, it gives an estimation of 40S fraction impurity. The ‘other’ category combines footprints and unaligned reads. (C) Representative footprint coverage profiles from normal Ribo-seq (gray) and 40S fraction (black). The dashed line marks the location of the *GCN4* uORFs. (D) Footprint length distribution in 80S and 40S fractions. Reads aligned to 18S rRNA are given as a size selection control for 40S fraction. Note: each distribution class has its own scale. (E) Cumulative coverage of footprints derived from 40S fraction. No normalization was applied prior to graph plotting. All genes regardless of their length were aligned by their start codon.

One of the advantages of Ribo-seq as well as other sequencing applications is its remarkable sensitivity and dynamic range. But that also requires us to deal with peculiarities in footprint coverage, which may be hard to interpret. Above, we presented an alternative view on 5′ UTR translation and, finally, would like to discuss an additional source of uORF-aligned footprints that does not come from an *in vivo* system. While studying translation in cell-free lysates, we examined to which extent the small ribosomal subunit can bind mRNA and produce footprints. There is not much evidence in the literature that a complex of mRNA with the small subunit is stable enough to withstand sucrose gradient fractionation. Nevertheless, we made an attempt to isolate such complexes and sequence footprints from them. Yeast lysates were treated with 1 mM puromycin in the presence of 500 mM KCl (Figure 5A). These conditions were previously reported to break apart ribosomal subunits (18). The small ribosomal subunit was separated by ultracentrifugation in a sucrose gradient; footprints were isolated and sequenced. The total number of footprints was small, just a fraction of a percent of all reads, coming from ribosomal rRNA contaminants (Figure 5B). Surprisingly, most of these footprints covered AUG start codons and were slightly shorter than normal footprints from 80S monosomes (Figure 5C–E). We cannot be sure the footprints come solely from the small subunit or also from reassembled full ribosomes that could still be present at low levels in our samples. Nevertheless, the finding demonstrates that dynamic binding of ribosome subunits to mRNA occurs in lysates. This process clearly involves 5′ UTRs, but not 3′ UTRs, and has to be taken into consideration when examining uORF translation.

In conclusion, we show that slow uptake of a translation inhibitor by live cells is the major cause of ribosomal accumulation at the beginning of coding in response to stress. We recommend avoiding pre-treatment of cell cultures with cycloheximide or other translation inhibitors, so that post-transcriptional regulation is not perturbed. Alternatively, if omitting the inhibitor is not an option, its concentration has to be as high as possible. During stress, the artifactual ribosomal distribution changes disproportionally, reflecting stress intensity rather than adaptation at the level of protein synthesis. Despite the fact that our experiments were conducted on yeast cells, the basic principles of cycloheximide diffusion into a cell should also apply to mammalian cells. Therefore, increasing concentrations of elongation inhibitors has to be tested prior to concluding on the elongation slowdown inflicted by stress or other experimental treatment. Our alternative point of view regarding uORF translation illustrates that Ribo-seq in its current state leaves too much room for speculation on the extent of their involvement in regulation. The problem is complicated by the short length of uORFs, that greatly decreases reproducibility of quantitative measurements presented as rpkm. The latter is apparent from various published Ribo-seq data sets—the full-sized protein ORFs display nearly perfect correlations between the replicates, whereas short uORFs show significant variation in expression. The Ribo-seq experimental design may need significant improvement to exclude ribosomal run-off and run-on in 5′ UTRs and to increase statistical power.

## Supplementary Material

Supplementary Data

## Data Availability

Sequencing data have been deposited to the Gene Expression Omnibus (http://www.ncbi.nlm.nih.gov/geo/) under accession number GSE59573
