# Supplementary material for: Translation inhibitors cause abnormalities in ribosome profiling experiments
Source: Nucleic Acids Res. 2014 Jul 23;42(17):e134. doi: 10.1093/nar/gku671 (PMC4176156; doi:10.1093/nar/gku671)
Supplement: Supplementary Data [file gku671_supplementary_data.zip › nar-00851-met-g-2014-File007.pdf]

Materials and methods (Extended)

Cells and treatments. Saccharomyces cerevisiae strain BY4741 was grown on YPD agar plates for several days prior to experiments. Unless otherwise stated, the day before the experiment cells were transferred to a 50 ml flask of YPD medium and grown overnight at 30 °C with shaking. A part of that culture was inoculated into 500 ml of fresh YPD at the initial OD_{600} = 0.025 and incubated at 30 °C with shaking until the OD_{600} reached 0.5-0.6. If cultures were designated for cyloheximide treatment, the drug was added at the end of any additional stress-inducing incubation. Immediately after drug addition, cells were harvested by vacuum filtration on 65 um PVDF filters (Millipore). It took exactly 5 min to collect the cells, which then were snap frozen in liquid nitrogen. If no drug treatment was needed, yeast cells were collected in the same manner, but filtration was initiated 5 min before the stress had to finish (see below).

Cycloheximide treatment. Concentrations of cycloheximide ranging from 1.56 to 10,000 μg/ml were used. We refer to 100 μg/ml as "x1", because it was used to inhibit protein translation in all other studies cited in this report. Therefore, other concentrations were marked as x1/64, x1/16, x1/4, x8, x100. For low concentrations, we used a stock solution in water. To achieve x8 concentration, we prepared the stock solution in DMSO. The highest possible concentration x100 was the most challenging. We first collected yeast cells by filtration, rapidly resuspended them in 5 ml of filtered YPD medium and added 5 ml of YPD with 20 mg/ml cycloheximide. This is the highest concentration possible considering drug solubility in water based solvents. Treatment with x100 concentration of cycloheximide was independently repeated two times and produced nearly identical traces of ribosome occupancy (Fig. S1).

Oxidative stress. To induce oxidative stress, cells were treated with 0.2 mM hydrogen peroxide. Cell culture was incubated for 30 min at 30 °C with shaking before harvesting.

Amino acid starvation. Yeast cells were cultured in SD medium instead of YPD. This medium consists of YNB (nitrogen source), CSM (amino acids plus other supplements) and glucose as a carbon source. When the OD_{600} reached 0.5-0.6 units, cells were harvested by filtration and transferred to a new flask of pre-warmed medium containing YNB and glucose only. After 20 min incubation, cells were harvested by filtration again and snap frozen. In some cases, leucine, histidine, methionine and uracil were supplemented individually (Figure 3A).

Heat shock. Upon reaching the OD_{600} of 0.5-0.6 in YPD, cells were harvested by filtration and transferred to a new flask of YPD preheated to 42 °C. Incubation lasted 20 min, then cells were harvested again by the same method and frozen.

Cell lysis and ribosome isolation. Frozen cell paste pellets were pulverized in a Mini Bead Beater (BioSpec) using stainless steel vials and chromium beads. To prevent yeast thawing, pulverization was done in multiple 10 seconds cycles where vials were repeatedly submerged to liquid nitrogen after each cycle. Therefore, the content of vials was kept frozen during pulverization. 1 ml of ice cold lysis buffer (20 mM Tris-HCl pH 8.0, 140 mM KCl, 5 mM $MgCl_2$, 1% Triton-X100, 100 μg/ml cycloheximide) was used to resuspend the pulverized cell powder. The lysates were clarified by centrifugation for 5 min. Absorbance at 260 nm was measured and 30 OD_{260} units were treated with 600 Units of RNAse I (Life Tech, Ambion) for 1 h at room temperature. The lysates were loaded on top of 10-50% sucrose gradient, prepared in the lysis buffer with no Triton. Ultracentrifugation in SW-41 Ti rotor for 3 h at 35000 rpm and 4 °C separated large ribosomal complexes from other cellular components. We used Brandel piercing system coupled with Biorad UV absorbance detector to collect monosome-containing fractions of sucrose gradients. Experiment with edeine was conducted similarly, with 2.5 μg/μl edeine in lysis buffer and sucrose buffers. Edeine was received from NCI repository (CAS

27656-72-0). Experiment with aurinticarboxylic acid (ATCA) was done slightly different: it cannot be added to the sucrose gradient buffers because of absorption at 254 nm. It also inhibits RNAse digestion. Therefore, yeast cells were lysed in a buffer with the addition of 0.2 mM aurintricarboxylic acid (Sigma) and then loaded onto a sucrose gradient with no ATCA. Polysomal and monosomal fractions were collected together; the buffer was substituted by the regular lysis buffer with no ATCA. After that, digestion by RNAse I was conducted as described above.

Isolation of a small ribosomal subunit. Frozen cell paste was cryogenically grinded as described above. Lysis was performed in 20 mM Tris-HCl pH 8.0, 500 mM KCl, 2.0 mM $MgCl_2$, 1% Triton-X100, 1 mM puromycin and 40 U/ml Superase-In. Lysate was incubated for 1 h at 37°C and then loaded on top of a 10-50% sucrose gradient in HiSalt Buffer (20 mM Tris-HCl, pH 7.5, 500 mM KCl, 5 mM MgCl). The peak corresponding to the small subunit was collected and footprints recovered as described below.

Footprint extraction. Sucrose fractions were concentrated with 100 kDa Amicon filter units (Millipore) to the volume of about 50-100 μl. The flow-through fraction was discarded. The volume of retentate was brought to 500 μl with release buffer (20 mM Tris-HCl pH 7.0, 6 mM EDTA, 40 U/ml Superase-In from Ambion) followed by 5 min incubation on ice. Samples were centrifuged for 5 min at 12,000 g and the flow-through fraction was collected this time. RNA footprints were purified by a single round of acid phenol chlorophorm extraction with subsequent RNA precipitation. Footprints from 40S fractions were isolated slightly different. After concentration with 100 kDa Amicon filter, the sample was diluted to 500 ul final volume with 20 mM Tris-HCl pH 8.0, 140mM KCl, 5.0 mM $MgCl_2$. Solution was treated with 250 U of RNAse I per 50 U of A_{280} for 15 min at room temperature, then spinned down on the same filter,

washed once with 20 mM Tris-HCl pH 8.0, 140mM KCl, 5.0 mM $MgCl_2$ and total RNA content was isolated from retentate by hot acid phenol chlorophorm.

Precipitation of RNA and DNA. The following components were added to the initial sample: 1/10 volume of ammonium acetate (Ambion), 5 μl of glycogen (5 mg/ml, Ambion) and 2.5 volumes of absolute ethanol. The mixture was incubated for 1 h at -20 °C, and nucleic acids were precipitated by a 15 min centrifugation at 20,000 g. This method was used to precipitate DNA and RNA after all enzymatic reactions.

Sequencing library preparation. Footprints were treated with T4 polynucleotide kinase (Thermo Scientific) for 1 h in 10 μl total reaction volume. RNA was loaded on a 15% TBE-Urea polyacrylamide gel (Invitrogen). The band corresponding to 25-32 nt was cut out of the gel, crushed with a disposable pestle (Kimble Chase), and RNA was eluted during a 3 h incubation at 37 °C in 0.3 ml of elution buffer (20 mM Tris-HCl pH 7.0, 2 mM EDTA, 1/10 volume of 3 M ammonium acetate, 40 U/ml Superase-In). Gel particles were eliminated by a Corning Costar spin-X 0.22 μm column. RNA was precipitated. Because various libraries were prepared over extended period of times, adapter sequences, reverse transcription primers and PCR primers were different. We used two sets of primers. The first set was designed to attach 6 nucleotide barcode to the 5' end of a footprint before sequencing; another set used indexes, which were not a part of a read. Primer sequences are listed in Supplementary Table 1 under Set #1 and Set #2.

100 ng of 3' adapter per sample were ligated by T4 RNA ligase 2 truncated KQ or K227Q (New England Biolabs). Reaction products were precipitated and reverse transcription was set up as follows. RNA pellet was dissolved in 11.5 μl of water, 4 pmol of reverse transcription primer were added along with 1 μl of dNTP mix (10 mM each). The mixture was incubated for 5 min at 65 °C, then chilled on ice. 2 μl of DTT, 4 μl of First Strand Buffer (refer to manufacturer`s

protocols for composition) were added along with 0.5 μl of SuperScript II and 0.5 μl Superase-In to the total volume of 20 μl. Reaction then continued for 30 min at 42 °C, 1 min at 65 °C, and 5 min at 80 °C. RNA was degraded in the presence of 80 mM NaOH at 95 °C for 30 min, then neutralized by the same amount of HCl. Reaction products were precipitated and run on 10% TBE-Urea polyacrylamide gel (Invitrogen), and the band corresponding to the transcriptase extended product was cut off. DNA was eluted as described above, precipitated and later used in CircLigase II reaction (20 μl total volume, according to manufacturer`s instructions, Epicentre). Products of ligation were used in the final PCR without additional precipitations or purifications. PCR was set up as follows: 10 pmol Reverse and Forward primers, 10 μl HF Buffer, 0.5 μl Phusion polymerase (New England Biolabs), 1-4 μl of ligation product, water up to 50 μl. PCR mix was subjected to 6-10 cycles of amplification (94 °C for 15 sec, 55 °C for 10 sec, 65 °C for 10 sec). Cycling was finalized for 2 min at 65 °C. PCR products were precipitated and run on 8% TBE polyacrylamide gel, and the band corresponding to the amplified products was cut off. DNA was eluted as described above, precipitated and sent for sequencing on an Illumina HiSeq 2000 platform.

Footprint alignment. Bowtie software v. 0.12.7 (1) was used to align footprints to *Saccharomyces cerevisiae* S288C genome, downloaded from SGD database with annotations. Custom Perl scripts were implemented to preprocess alignment files and plot ribosomal occupancy.

Ribosomal occupancy distribution plot. We selected all single exon genes longer than 1000 nucleotides expressed at rpkm > 30. The selection of these criteria allows accounting for ~2300 genes. The parameter could be set differently, for instance rpkm > 10 – that would not change the conclusions. They were aligned by start codon and coverage at every nucleotide position of

every gene was averaged. The plot covers 1000 nucleotides within reading frame plus 50 nucleotides upstream of the start codon. The average coverage density of the last 300 nucleotides (within the selected thousand) was used to normalize ribosome occupancy so that each profile approached the value of 1.0. We used the entire footprint sequence to calculate the coverage, therefore the profile line appears smooth. Alternatively, only 5′ or 3′ ends of footprints could be used, then the profile would be more irregular.

Aligning footprints to 5′ UTR. We used a list of 5′ UTR coordinates from (2). Additionally, for each gene we added up to 50 nt upstream of the start codon if the record in (2) annotated a shorter sequence. An extra check was done to ensure the absence of overlaps between 5′ UTR of a gene with a 3′ end of an adjacently located gene.

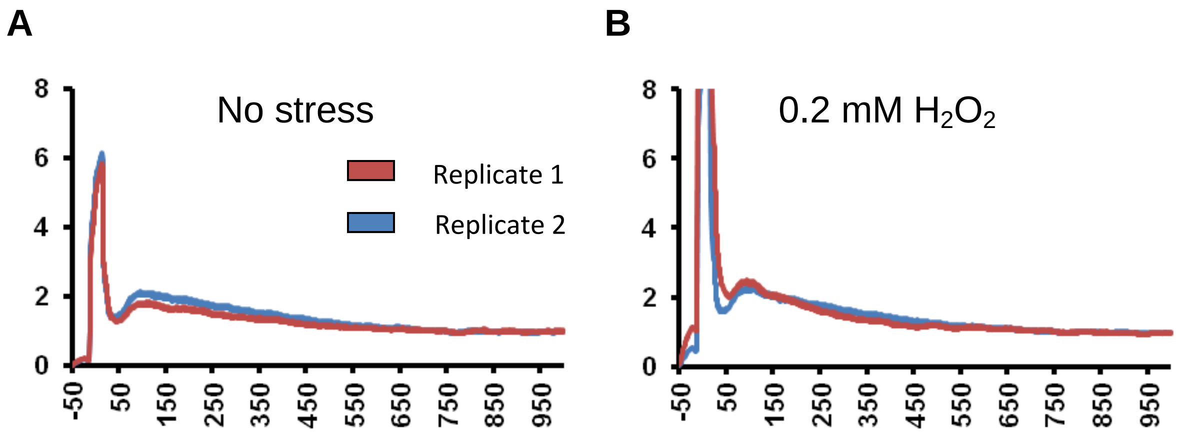

Supplementary Figure S1. Traces of ribosome occupancy for x100 concentration of cycloheximide were reproduced in two independent experiments. (A) No stress. (B) 0.2 mM hydrogen peroxide induced oxidative stress**.**

Supplementary Table 1. Sequences of primers and adapters used for library preparation.

**Set #1**	
3' adapter	rAppCTGTAGGCACCATCAAT/3ddC/
RT-primers (barcoded)	pCGTGATGATCGTCGGACTGTAGAACTCTGAACCTGTCGGTGGTCGCCGTATCATT/iSp18/CAAGCAGAAGACGGCATACGAATTGATGGTGCCTACAG
	pTGGTCAGATCGTCGGACTGTAGAACTCTGAACCTGTCGGTGGTCGCCGTATCATT/iSp18/CAAGCAGAAGACGGCATACGAATTGATGGTGCCTACAG
	pATTGGCGATCGTCGGACTGTAGAACTCTGAACCTGTCGGTGGTCGCCGTATCATT/iSp18/CAAGCAGAAGACGGCATACGAATTGATGGTGCCTACAG
	pACATCGGATCGTCGGACTGTAGAACTCTGAACCTGTCGGTGGTCGCCGTATCATT/iSp18/CAAGCAGAAGACGGCATACGAATTGATGGTGCCTACAG
	pCACTGTGATCGTCGGACTGTAGAACTCTGAACCTGTCGGTGGTCGCCGTATCATT/iSp18/CAAGCAGAAGACGGCATACGAATTGATGGTGCCTACAG
	pGCCTAAGATCGTCGGACTGTAGAACTCTGAACCTGTCGGTGGTCGCCGTATCATT/iSp18/CAAGCAGAAGACGGCATACGAATTGATGGTGCCTACAG
	pTCAAGTGATCGTCGGACTGTAGAACTCTGAACCTGTCGGTGGTCGCCGTATCATT/iSp18/CAAGCAGAAGACGGCATACGAATTGATGGTGCCTACAG
	pGATCTGGATCGTCGGACTGTAGAACTCTGAACCTGTCGGTGGTCGCCGTATCATT/iSp18/CAAGCAGAAGACGGCATACGAATTGATGGTGCCTACAG
	pAAGCTAGATCGTCGGACTGTAGAACTCTGAACCTGTCGGTGGTCGCCGTATCATT/iSp18/CAAGCAGAAGACGGCATACGAATTGATGGTGCCTACAG
	pGTAGCCGATCGTCGGACTGTAGAACTCTGAACCTGTCGGTGGTCGCCGTATCATT/iSp18/CAAGCAGAAGACGGCATACGAATTGATGGTGCCTACAG
	pTACAAGGATCGTCGGACTGTAGAACTCTGAACCTGTCGGTGGTCGCCGTATCATT/iSp18/CAAGCAGAAGACGGCATACGAATTGATGGTGCCTACAG
	pCTGATCGATCGTCGGACTGTAGAACTCTGAACCTGTCGGTGGTCGCCGTATCATT/iSp18/CAAGCAGAAGACGGCATACGAATTGATGGTGCCTACAG
PCR forward	CAAGCAGAAGACGGCATACGA
PCR reverse	AATGATACGGCGACCACCGA
**Set #2**	
3' adapter	rAppAGATCGGAAGAGCACACGTCT/3ddC/
RT-primer	pGATCGTCGGACTGTAGAACTCTGAACCTGTCGGTGGTCGCCGTATCATT/iSp18/GTGACTGGAGTTCAGACGTGTGCTCTTCCGATCT
PCR forward	AATGATACGGCGACCACCGACAGGTTCAGAGTTCTACAGTCCGACGATC
PCR reverse primers (indexed)	CAAGCAGAAGACGGCATACGAGAT**CGTGAT**GTGACTGGAGTTCAGACGTGTGCTCTTCCGATCT
	CAAGCAGAAGACGGCATACGAGAT**ACATCG**GTGACTGGAGTTCAGACGTGTGCTCTTCCGATCT
	CAAGCAGAAGACGGCATACGAGAT**GCCTAAG**TGACTGGAGTTCAGACGTGTGCTCTTCCGATCT
	CAAGCAGAAGACGGCATACGAGAT**TGGTCA**GTGACTGGAGTTCAGACGTGTGCTCTTCCGATCT
	CAAGCAGAAGACGGCATACGAGAT**CACTGT**GTGACTGGAGTTCAGACGTGTGCTCTTCCGATCT
	CAAGCAGAAGACGGCATACGAGAT**ATTGGC**GTGACTGGAGTTCAGACGTGTGCTCTTCCGATCT
	CAAGCAGAAGACGGCATACGAGAT**GATCTG**GTGACTGGAGTTCAGACGTGTGCTCTTCCGATCT
	CAAGCAGAAGACGGCATACGAGAT**TCAAGT**GTGACTGGAGTTCAGACGTGTGCTCTTCCGATCT
	CAAGCAGAAGACGGCATACGAGAT**CTGATC**GTGACTGGAGTTCAGACGTGTGCTCTTCCGATC

	T
	CAAGCAGAAGACGGCATACGAGAT**AAGCTA**GTGACTGGAGTTCAGACGTGTGCTCTTCCGATC<br>T
	CAAGCAGAAGACGGCATACGAGAT**AAGCTA**GTGACTGGAGTTCAGACGTGTGCTCTTCCGATC<br>T
	CAAGCAGAAGACGGCATACGAGAT**TACAAG**GTGACTGGAGTTCAGACGTGTGCTCTTCCGATC<br>T

References

1. Langmead, B., Trapnell, C., Pop, M. and Salzberg, S.L. (2009) Ultrafast and memory-efficient alignment of short DNA sequences to the human genome. *Genome Biol*, **10**, R25.
2. Nagalakshmi, U., Wang, Z., Waern, K., Shou, C., Raha, D., Gerstein, M. and Snyder, M. (2008) The transcriptional landscape of the yeast genome defined by RNA sequencing. *Science*, **320**, 1344-1349.
